# Novel Characterization and Live Imaging of Schlemm's Canal Expressing Prox-1

**DOI:** 10.1371/journal.pone.0098245

**Published:** 2014-05-14

**Authors:** Tan N. Truong, Hannah Li, Young-Kwon Hong, Lu Chen

**Affiliations:** 1 Graduate Group in Vision Science, University of California, Berkeley, California, United States of America; 2 Center for Eye Disease and Development, Program in Vision Science and School of Optometry, University of California, Berkeley, California, United States of America; 3 Department of Surgery and Department of Biochemistry and Molecular Biology, Norris Comprehensive Cancer Center, Keck School of Medicine, University of Southern California, Los Angeles, California, United States of America; 4 The Francis I. Proctor Foundation for Research in Ophthalmology, University of California San Francisco, San Francisco, California, United States of America; 5 Schepens Eye Research Institute, Massachusetts Eye and Ear Infirmary, Harvard Medical School, Boston, Massachusetts, United States of America; Casey Eye Institute, United States of America

## Abstract

Schlemm's canal is an important structure of the conventional aqueous humor outflow pathway and is critically involved in regulating the intraocular pressure. In this study, we report a novel finding that prospero homeobox protein 1 (Prox-1), the master control gene for lymphatic development, is expressed in Schlemm's canal. Moreover, we provide a novel in vivo method of visualizing Schlemm's canal using a transgenic mouse model of Prox-1-green fluorescent protein (GFP). The anatomical location of Prox-1^+^ Schlemm's canal was further confirmed by in vivo gonioscopic examination and ex vivo immunohistochemical analysis. Additionally, we show that the Schlemm's canal is distinguishable from typical lymphatic vessels by lack of lymphatic vessel endothelial hyaluronan receptor (LYVE-1) expression and absence of apparent sprouting reaction when inflammatory lymphangiogenesis occurred in the cornea. Taken together, our findings offer new insights into Schlemm's canal and provide a new experimental model for live imaging of this critical structure to help further our understanding of the aqueous humor outflow. This may lead to new avenues toward the development of novel therapeutic intervention for relevant diseases, most notably glaucoma.

## Introduction

Schlemm's canal is a circumferential channel located at the iridocorneal angle in the ocular anterior chamber. It is part of the conventional aqueous humor outflow system of the anterior chamber, which accounts for 70–90% of the total aqueous humor that drains out of the eye. The endothelial cell lining of Schlemm's canal is one of the primary sites of resistance to aqueous humor drainage and is a major determinant of intraocular pressure [1]. Intraocular pressure when elevated can often lead to glaucoma, a disease affecting approximately 60 million people worldwide and is the second leading cause of blindness globally [2]. However, the particular contribution and exact mechanisms by which this continuous endothelium monolayer of Schlemm's canal resists aqueous humor outflow still remain largely unclear in both normal and glaucomatous eyes. It is therefore essential to enhance our knowledge on this important structure, which is a crucial prerequisite for developing new therapeutic strategies.

The cellular features and specialized functions of the canal's endothelium have recently been speculated as being both lymphatic and blood vascular in nature [3]. It was reported earlier that the Schlemm's canal endothelia are derived from a vascular origin and retain some properties of blood vessels [4], [5]. Similarly, it is reported that the endothelial cell origins of the lymphatic vessels stem from the venous vasculature, and the transcription factor, prospero-related homeobox 1 (Prox-1), is largely responsible for the induction of lymphatic endothelial cell phenotype during development [6], [7]. Interestingly, there has been recent evidence to suggest that ocular lymphatics contribute to drainage of aqueous humor from the eye [8], [9], [10]. However, the physical and physiological relationship between Schlemm's canal and the lymphatic system has yet to be thoroughly assessed. To date, there has been no report linking Prox-1 to the Schlemm's canal.

Lymphatic research has progressed rapidly in recent years and the importance of the lymphatic system is now well-recognized in health and disease [11], [12]. The lymphatic system is comprised of an extensive network of vessels that penetrates through most tissues of the body and carries out important functions including tissue fluid homeostasis, immune surveillance, as well as fat absorption. Since a large portion of the fluids leaked out of the blood capillaries in the peripheral tissues are returned back to the blood circulation via the lymphatic system, lymphatic dysfunction can lead to drainage disorders such as tissue swelling or lymphedema. Prox-1, the master control gene of lymphatic development, along with several other lymphatic endothelial cell markers, such as lymphatic vessel endothelial hyaluronic acid receptor-1 (LYVE-1) and vascular endothelial growth factor-3 (VEGFR-3), have been extensively used in lymphatic research to allow for identification and exploration into the active growth of lymphatic vessels during both developmental and pathological processes [6], [13], [14].

In this study, we performed mouse live imaging using our newly developed advanced imaging system and a transgenic mouse model of Prox-1-green fluorescent protein (GFP) [15], [16]. The expression of GFP under the Prox-1 promoter in the transgenic mice allowed for direct and convenient visualization of lymphatic vasculatures in vivo. In doing so, we discovered that besides limbal lymphatics, Prox-1 was expressed on a previously unidentified structure at the iridocorneal angle, which was Schlemm's canal. The anatomical location of the Prox-1^+^ Schlemm's canal was further confirmed by in vivo gonioscopic examination as well as ex vivo immunohistochemical analysis. Moreover, we found that the highly recognizable Prox-1^+^ Schlemm's canal was distinguishable from typical lymphatic vessels by lack of LYVE-1 expression and absence of apparent sprouting reaction when inflammatory lymphangiogenesis was induced from limbal lymphatics.

## Methods

### Mice and anesthesia

Transgenic Prox-1-GFP mice of FVB/N or C57BL/6 background and wildtype mice (adult mice ≥ 12 weeks of age and postnatal mice of 3 weeks of age) were used in the experiments [15]. The Prox1-GFP BAC construct was created by inserting the GFP-coding sequences under the Prox-1 promoter in a Prox-1-harboring BAC through homologous recombination by the GENSAT researchers [17]. This BAC contains a mouse genomic contig harboring all regulatory elements for Prox-1 expression and it has been shown that the Prox-1-GFP mice faithfully recapitulate Prox-1 expression in lymphatic vessels with no morphologic alternation [15]. All mice were treated according to ARVO Statement for the Use of Animals in Ophthalmic and Vision Research, and all protocols were approved by the Animal Care and Use Committee of University of Southern California or University of California, Berkeley. Local anesthesia with topical 0.5% proparacaine hydrochloride ophthalmic solution (Bausch & Laumb, Rochester, NY) and general anesthesia using a mixture of ketamine, xylazine, and acepromazine (50 mg, 10 mg, and 1 mg/kg body weight, respectively) were administered for each surgical procedure.

### In vivo imaging and gonioscopic examination

In vivo imaging of mice was performed as we reported recently [16]. Digital brightfield and fluorescent micrographs of the Prox-1^+^ structures were taken using an advanced and customized imaging system consisting of Zeiss Axio zoom V.16 (Carl Zeiss AG, Gottingen, Germany) and an adjustable eye and head stage holder. This non-contact imaging system with large stereomicroscope field of view allowed for imaging of whole cornea in separate brightfield and fluorescence contrast. Z-stack image captures were processed with Helicon Focus imaging software (Heliconsoft Ltd.) to obtain extended focus images with increased depth of field. Additionally, utilizing this system in conjunction with a specialized 2.0 mm mouse gonioprism (Ocular Instruments, Bellevue, WA) allowed for in vivo non-invasive brightfield and fluorescent microscopic imaging of the iridocorneal angle of the anterior chamber [18]. The experiment was repeated at least twice with six mice included in the study.

### Electron microscopy

Eyeballs were sectioned with anterior angle intact and fixed in 2% glutaraldehyde 0.1M sodium cacodulate buffer. Tissues were then post-fixed with 1% osmium tetroxide followed by incubation with 0.5% aqueous uranyl acetate. Samples were dehydrated with series of increasing acetone concentration followed by resin embedding. Tissue blocks from 3 to 5 different locations were sectioned and mounted on copper grids. After staining with uranyl acetate and led citrate, samples were imaged with Tecnai 12 transmission electron microscope (FEI, Hillsboro, OR).

### Suture-induced inflammatory lymphangiogenesis

The standard suture placement model with 11-0 nylon sutures (AROSurgical, Newport Beach, CA) was used to induce corneal inflammatory lymphangiogenesis, as reported previously [19], [20], [21]. Sutures were placed intrastromally without penetrating into the anterior chamber. The experiment was repeated twice with four Prox-1-GFP transgenic mice included in the study.

### Immunohistochemical assays and epifluorescent and confocal microscopy

The experiments were performed similarly as reported previously [19], [20]. Briefly, 1% paraformaldehyde or acetone was used for tissue fixation. Cryosections of eyeballs or whole-mount full thickness tissues were blocked in 10% donkey serum and immunostained with one or two of the following antibodies: LYVE-1 (Abcam, Cambridge, MA), alpha smooth muscle actin (αSMA, Abcam), CD31 (BD Pharmingen, San Jose, CA), Prox-1 (AngioBio, Del Mar, CA), and VE-cadherin (Abcam). Samples were visualized by subsequent staining with FITC and/or Cy-3 conjugated donkey anti-rabbit and/or anti-rat antibodies (Jackson ImmunoResearch Laboratories, West Grove, PA). Samples were covered with Vector Shield mounting medium (Vector Laboratories, Burlingame, CA) and examined by an AxioImager M1 epifluorescence deconvolution microscope with AxioVision 4.8 software (Carl Zeiss AG, Göttingen, Germany). In addition, whole-mount corneal samples together with the limbal area were evaluated with a LSM 780 NLO AxioExaminer confocal microscope (Carl Zeiss AG), and z-stack images were processed with NIH Image-J and Imaris processing software to generate three-dimensional images and videos (Bitplane, Zurich, Switzerland) [22]. The experiment was repeated at least three times with eight Prox-1 transgenic and six wildtype eyes from adult mice. An additional six eyes at the postnatal age of 3 weeks were used for developmental analysis.

## Results

### High Prox-1 GFP expression at the iridocorneal angle

**Figure 1:**
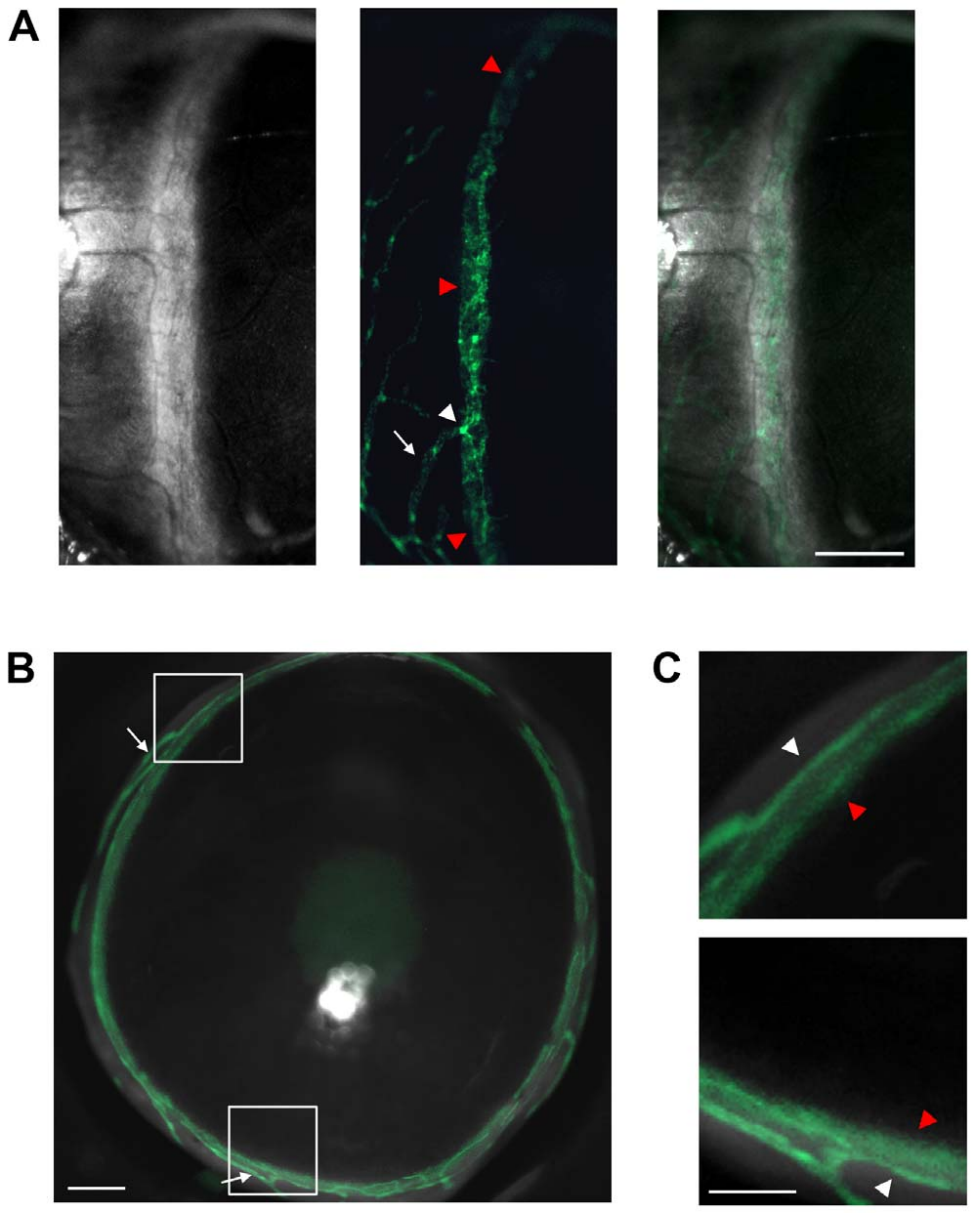
Brightfield and fluorescent microscopic evaluation of the anterior segment of adult Prox-1 GFP transgenic mouse eye. (A) In vivo profile view. Left, brightfield; Middle, green fluorescence; Right, merged image. Prox-1^+^ conjunctival (white arrows) and limbal vessels (white arrowheads) are visible. A broad, continuous structure (red arrowheads) expressing Prox-1 was also detected near the limbal area. (B) Frontal view of an ex vivo Prox-1 GFP mouse cornea together with the limbal area. White arrows: Prox-1^+^ conjunctival lymphatic vessels. (C) Enlarged view of boxed regions in (B). White arrowheads: Prox-1^+^ limbal lymphatic vessels. Red arrowheads: Prox-1^+^ structure located deeper at the iridocorneal angle of the anterior chamber. Green: Prox-1. Scale bars, 500 µm (A); 200 µm (B); 100 µm (C).

Our initial in vivo microscopic survey of the adult Prox-1-GFP transgenic mouse eye revealed a large continuous band of Prox-1 expression near the corneal limbus (Figure 1A). The in vivo diameter of this band was larger than that of the limbal or conjunctival lymphatic vessels. Further ex vivo examination confirmed there were two distinct Prox-1^+^ structures around the limbal area (Figure 1B and 1C). The first and more superficial structure belonged to limbal lymphatics at the ocular surface. The second and deeper structure ran along the iridocorneal angle where the cornea and the iris met. Since both trabecular meshwork and Schlemm's canal are anatomically located at this angle, our findings suggested that this deeper Prox-1^+^ structure was most likely either the trabecular meshwork or Schlemm's canal.

### Gonioscopic examination of the iridocorneal angle

**Figure 2:**
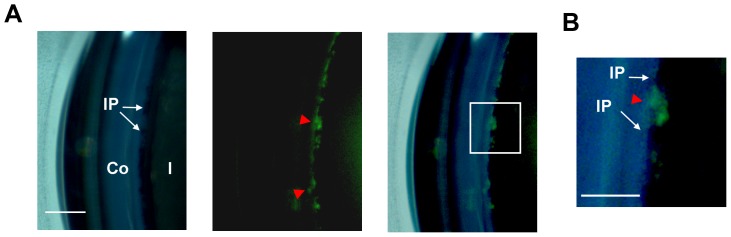
In vivo gonioscopic evaluation of adult Prox-1 GFP mouse eye. (**A**) Gonioscopic view of the iridocorneal angle of the anterior chamber. Direct view of the angle showing the Prox-1^+^ structure (red arrowheads) in areas where pigmented iris processes (white arrows) were absent. Left, brightfield; Middle, green fluorescence; Right panels: merged image. (**B**) Enlarged image of boxed area in (B). Green: Prox-1. Scale bars, 200 µm (A, B); 100 µm (C). Co, cornea; Cj, conjunctiva; I, iris; IP: iris processes.

As shown in Figure 2, additional evaluation of the iridocorneal angle was performed with in vivo gonioscopic examination using a specialized mouse gonioprism. As shown in Figure 2A and 2B, views through the peripheral mirror lens allowed for a direct and unobstructed view of the iridocorneal angle. Prox-1^+^ expression was detected at the angle in areas absent of iris processes (IP), which were bands of pigmented tissue extending from the iris and bridging over the angle structure.

### Electron microscopic examination of the Schlemm's canal

**Figure 3:**
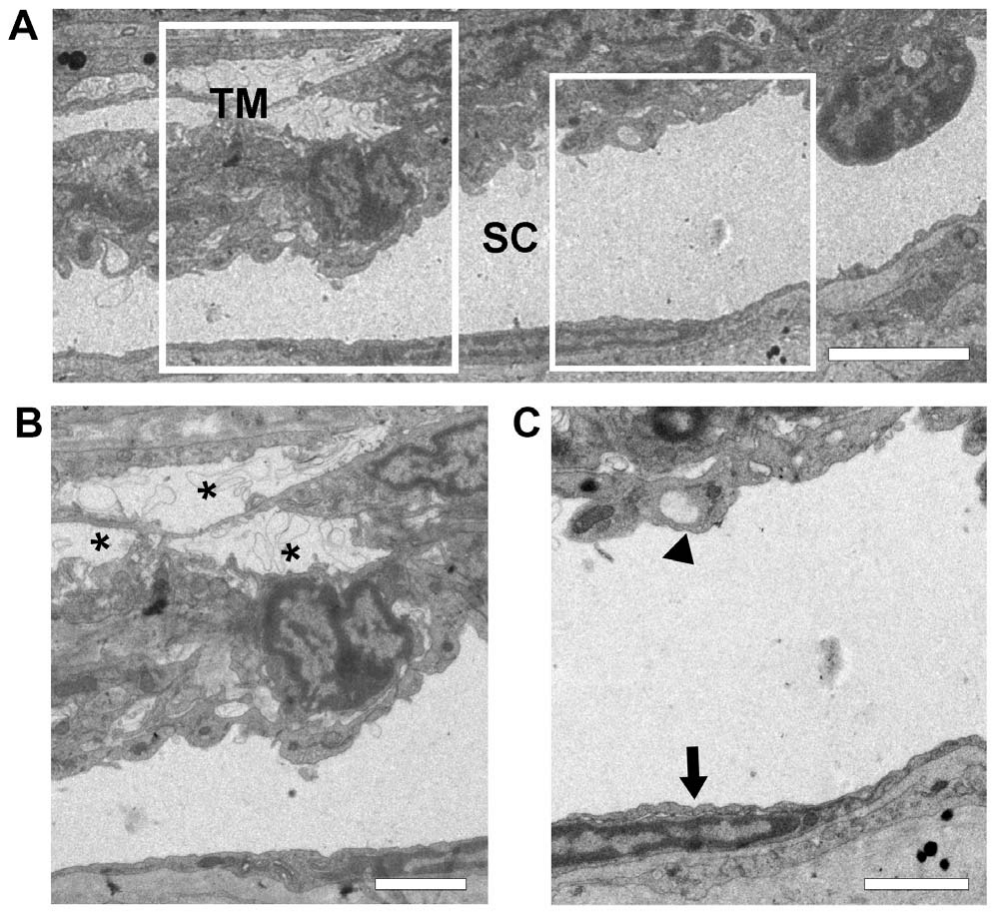
Ultrastructure electron micrographs of Schelmm's canal in Prox-1 GFP mice. (**A**) Representative image showing normal morphology of Schlemm's canal (SC) and nearby trabecular meshwork (TM) in adult mice. (B) Magnified left boxed region in (A) of TM with characteristic intertrabecular spaces (asterisks). (C) Magnified right boxed region in (A) showing outer wall of Schlemm's canal lined with endothelial cells (arrow). A typical giant vacuole (arrowhead) was observed on the inner wall of Schlemm's canal. Scale bars, 4 µm (A); 2 µm (B, C).

It has been shown that the Prox-1-GFP mice faithfully recapitulate the expression pattern of Prox-1 in cells and structures without causing morphological change [15]. This is also confirmed by our electron microscopic examination of the Prox-1-GFP mice where normal and typical morphology of Schlemm's canal and trabecular meshwork was observed, as shown in Figure 3. While the outer wall of Schlemm's canal was lined with a monolayer of endothelial cells, typical giant vacuoles were seen on the inner wall protruding from the trabecular meshwork where characteristic intertrabecular spaces were observed.

### Identification of Schlemm's canal by immunohistochemical analysis

**Figure 4:**
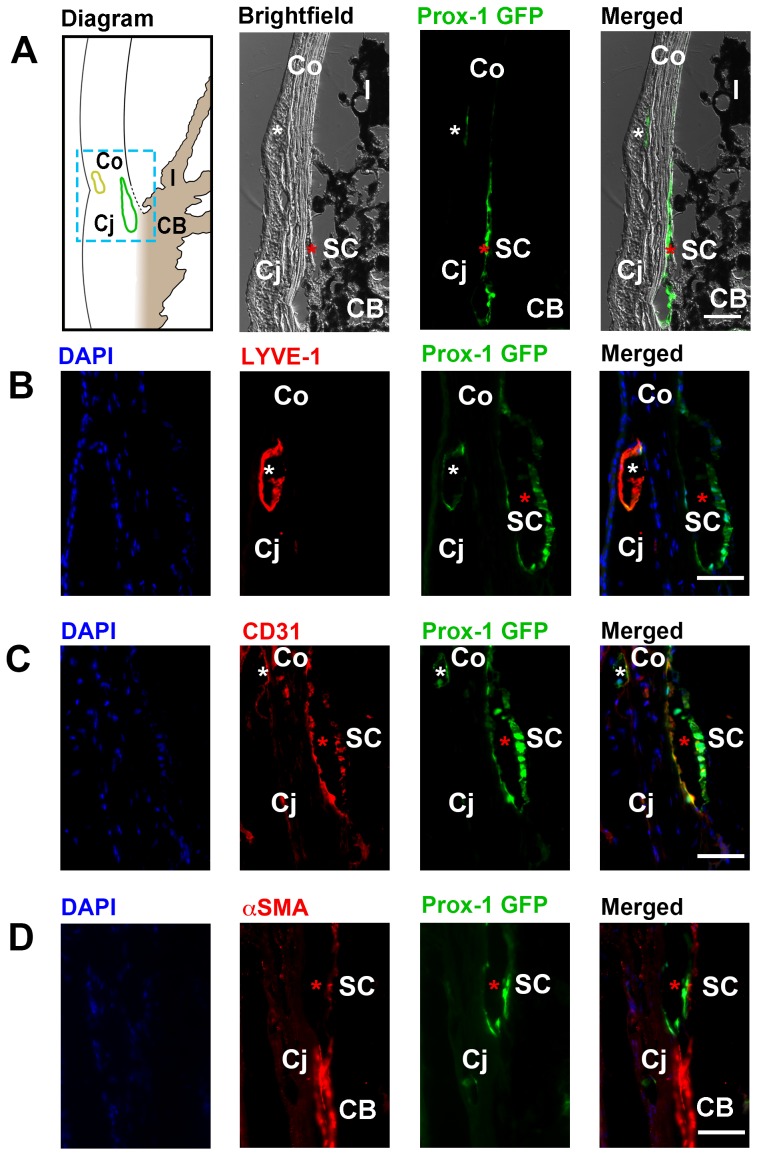
Cross-sectional immunohistochemical analysis of the iridocorneal angle of adult Prox-1-GFP mice. (**A**) Left panel, illustrative diagram of anterior chamber angle showing location of Schlemm's canal (green) at the corneoscleral junction. Yellow, limbal lymphatic vessels; Middle to right panels: brightfield, green fluorescent, and merged micrographs corresponding to the boxed region of interest in the diagram. The boxed region in the diagram is on different scale for illustrative purpose. White asterisks: limbal lymphatics; Red asterisks: Schlemm's canal. (**B**) Representative images showing the LYVE-1^+^Prox-1^+^ limbal lymphatic vessel (white asterisk) located between the cornea and conjunctiva, and the LYVE-1^−^Prox1^+^ Schlemm's canal (red asterisk) located nearby. Blue: DAPI for nuclear staining; Red: LYVE-1; Green: Prox-1. (**C**) Representative images showing both limbal lymphatics (white asterisk) and the Schlemm's canal (red asterisk) expressed CD31, a panendothelial cell marker. Blue: DAPI; Red: CD31; Green: Prox-1. (**D**) Representative images showing αSMA was not expressed on the Prox-1^+^ Schlemm's canal (red asterisk), but on adjacent positive control tissue of the ciliary body. DAPI: blue; Red: αSMA; Green: Prox-1. Scale bars, 50 µm (A–D). SC, Schlemm's canal; Co, cornea; Cj, conjunctiva; I, iris; CB: ciliary body.

**Figure 5:**
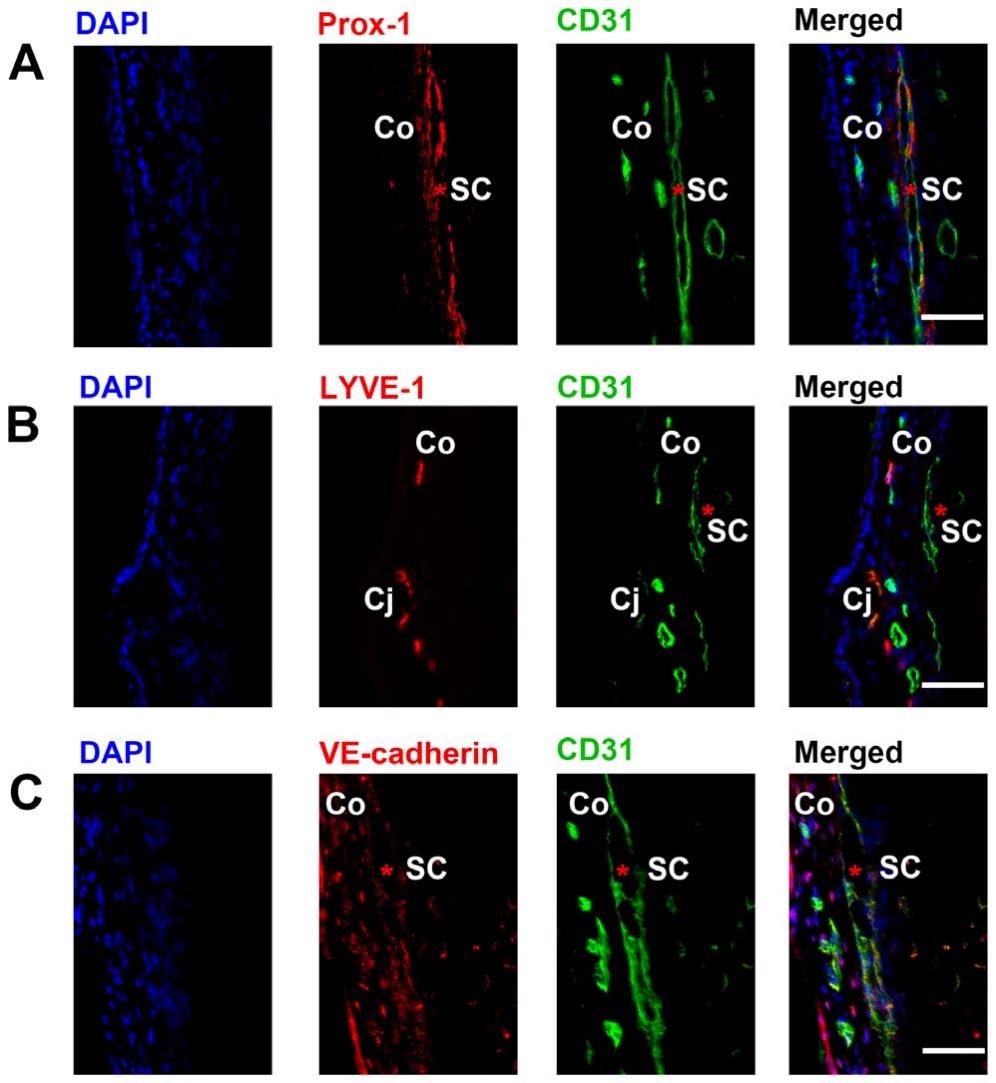
Cross-sectional immunohistochemical analysis of the iridocorneal angle of adult wildtype mice. (A) Representative images showing that Schlemm's canal (red asterisk) is Prox-1^+^ and CD31^+^. Blue: DAPI for nuclear staining; Red: Prox-1; Green: CD31. (**B**) Representative images showing CD31^+^ Schlemm's canal (red asterisk) is LYVE-1^−^. Blue: DAPI; Red: LYVE-1; Green: CD31^+^. (**C**) Representative images showing VE-cadherin was expressed on the CD31^+^ Schlemm's canal (red asterisk). DAPI: blue; Red: VE-cadherin; Green: CD31. Scale bars, 50 µm (A–C). SC, Sclemm's canal; Co, cornea; Cj, conjunctiva.

To further identify the Prox-1^+^ structure at the iridocorneal angle, we next performed a series of cross-sectional immunohistochemical assays using specific antibodies against CD31, LYVE-1, and αSMA. As illustrated by a schematic diagram of the structures surrounding the iridocorneal angle and cross-sectional views of the Prox-1-GFP mice, our initial examination under both light and fluorescent microscopes showed that Prox-1 was expressed on limbal lymphatics and the iridocorneal angle where Schlemm's canal was located (Figure 4A). Our additional evaluation by a series of immunohistochemical assays further confirmed that the Prox-1^+^ angle structure was Schlemm's canal. It contained a typical central lumen in the shape of an elongated ellipse and expressed CD31 (Figure 4C), a known panendothelial cell marker present in Schlemm's canal [23]. Moreover, this structure was negative for αSMA expression, which was detected in the ciliary muscle adjacent to the canal (Figure 4D). In contrast to the high expression of LYVE-1 on Prox-1^+^ limbal lymphatics, LYVE-1 was not detected on the Prox-1^+^ Schlemm's canal (Figure 4B). The presence of this Prox-1^+^CD31^+^ but LYVE-1^-^ Schlemm's canal was also confirmed in wildtype/non Prox-1-GFP mice, as shown in Figure 5. This structure also expressed vascular endothelial (VE) cadherin, as reported previously [23]. Additionally, we have confirmed the expression pattern of Prox-1^+^CD31^+^ but LYVE-1^−^ in the Schlemm's canal at a developmental stage (3 weeks postnatal) in both Prox-1-GFP and wildtype mice (Figure S1 and S2) [24].

**Figure 6:**
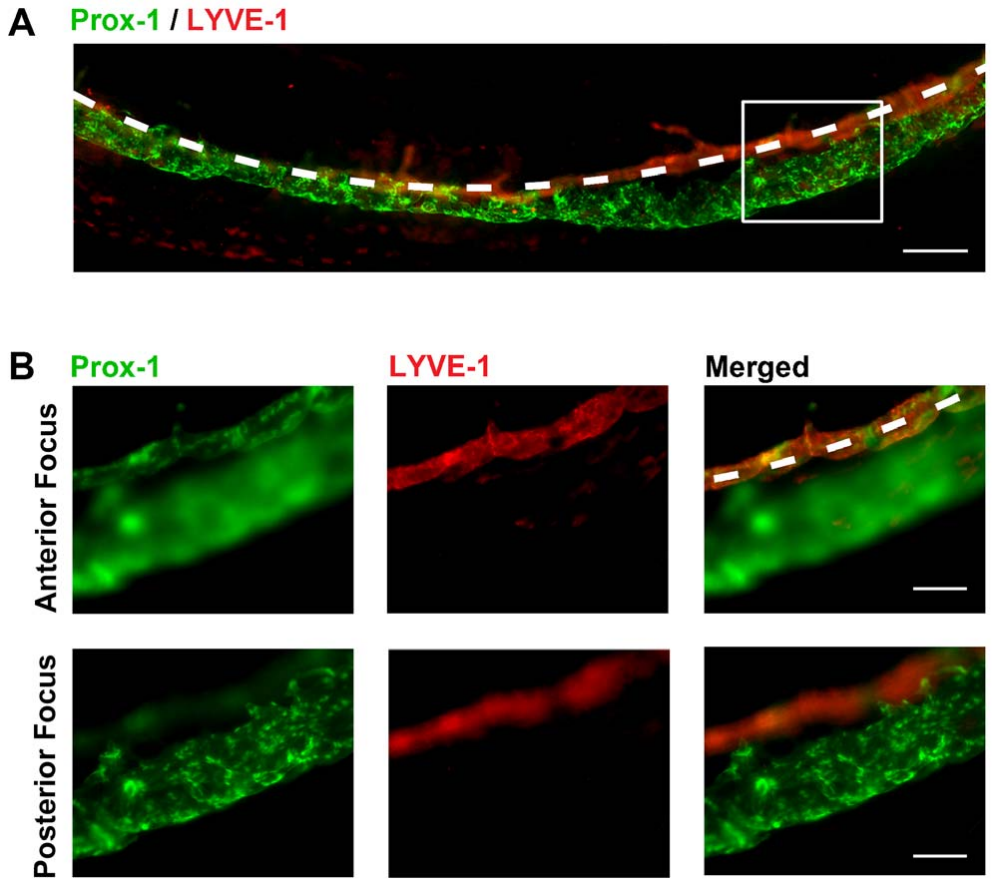
Whole-mount immunohistochemical analysis of the Prox-1^+^ structures in normal adult mice. (**A**) Micrograph of approximately 1/3 of the limbus (demarcated by dashed white line) showing the location and continuity of Prox-1^+^LYVE-1^−^ Schlemm's canal. (**B**) Magnified view of boxed area in (A). Top panel micrographs were taken with microscope objective anteriorly focused on Prox-1^+^LYVE-1^+^ limbal lymphatic vessels (demarcated by dashed white line). The posterior Prox-1^+^LYVE-1^−^ Schlemm's canal was defocused. Bottom panel micrographs were taken with microscope objective posteriorly focused on the Prox-1^+^LYVE-1^−^ Schlemm's canal. The limbal lymphatic vessel was defocused. Red: LYVE-1; Green: Prox-1. Scale bars, 200 µm (A); 100 µm (B).

The structural morphology of Schlemm's canal was further examined by whole-mount tissue immunohistochemical analysis. As shown in Figure 6A, the Prox-1^+^LYVE-1^−^ Schlemm's canal was readily distinguishable from the Prox-1^+^LYVE-1^+^ limbal vessels (Figure 6A). The diameter of the Schlemm's canal varied along the circumference of the angle. Even at the thinnest portion of the canal, its diameter was equal to or greater than that of the limbal lymphatics. This confirmed our noted in vivo observation that the annular Prox-1^+^ angle structure, now identified as Schlemm's canal, had a larger diameter relative to conjunctival or limbal lymphatic vessels. It was also confirmed that the limbal lymphatics were clearly in focus and Schlemm's canal was defocused when focusing the microscope objective anteriorly at the ocular surface where LYVE-1+ non-endothelial cells were also present [12], [25]. In contrast, the exact opposite was the case when the objective was focused more posteriorly with Schlemm's canal in clear focus while the limbal vessels were defocused (Figure 6B).

### Schlemm's canal shows no apparent sprouting reaction during inflammatory lymphangiogenesis from limbal vessels

**Figure 7:**
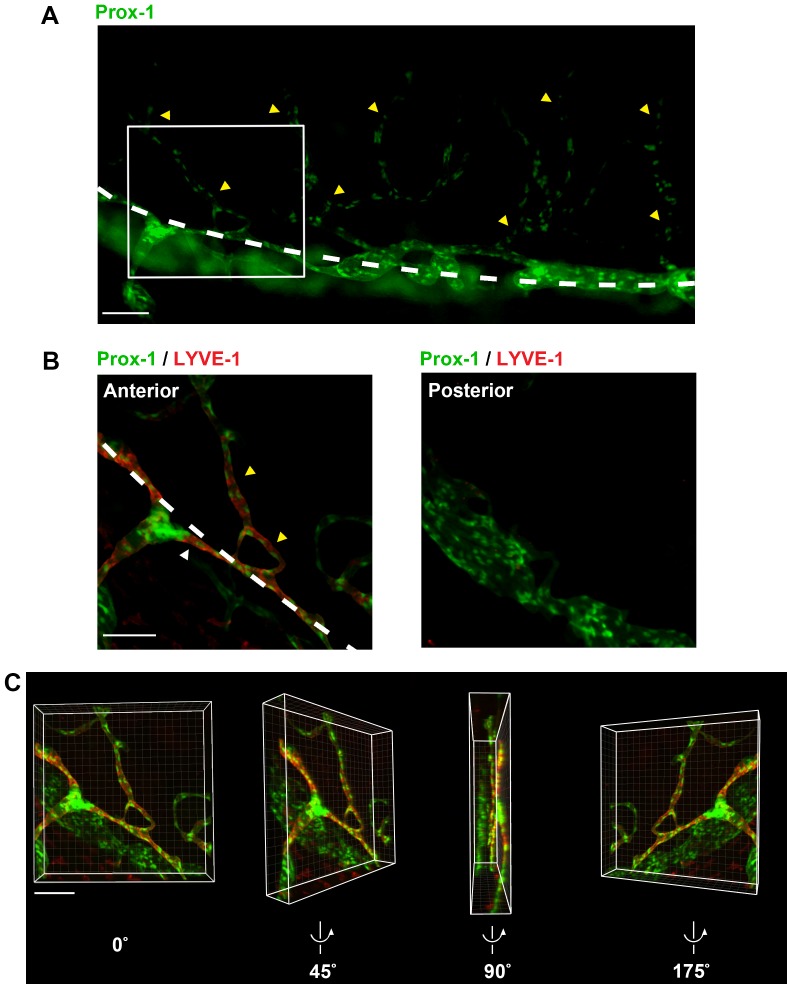
Differential responses of the Prox-1^+^ structures during corneal inflammatory lymphangiogenesis. (**A**) Epifluorescent micrograph showing sprouting lymphatic vessels into the inflamed cornea 2 weeks after suture placement. White dashed line: the limbus. Yellow arrowheads: newly formed lymphatics emanating from the limbus and growing into central cornea. (**B**) Confocal micrographs of boxed region in (A) showing differential sprouting reaction of the two Prox-1^+^ structures located at the limbus and the angle, respectively. Left panel: anterior projection of the confocal z-stack showing Prox-1^+^LYVE-1^+^ limbal (white arrowhead) and newly formed corneal lymphatic vessels (yellow arrowheads). Dashed white line: the limbus. Right panel: posterior projection of the confocal z-stack where Prox-1^+^ LYVE-1^−^Schlemm's canal was located. No apparent sprouting reaction, or newly formed vessels were detected. (**C**) Images captured from 3-dimensional rendering of (B) with 0°, 45°, 90°, and 175° rotation around the central vertical axis. Green: Prox-1; Red: LYVE-1. Scale bar: 100 µm (A–C).

To further distinguish the two Prox-1^+^ structures we identified at the limbus and the iridocorneal angle, we assessed sprouting reaction using the standard suture-induced inflammatory lymphangiogenesis model. Our results showed that after suture placement, the Prox-1^+^ limbal structure generated new lymphatic branches (Figure S3). The newly formed lymphatics expressed LYVE-1 (Figure 7A; 7B, left panel). In contrast, no apparent new branches were detected from the Schlemm's canal, which remained LYVE-1 negative as well (Figure 7B, right panel). This observation was also confirmed with three-dimensional rendering of confocal z-stacks (Figure 7C, Video S1).

## Discussion

To our knowledge, this study provides the first evidence showing the high expression of the lymphatic marker Prox-1 on Schlemm's canal endothelium. It also provides a new method for in vivo visualization of the canal in its entirety. Moreover, we have shown that the Schlemm's canal is distinguishable from typical lymphatic endothelium by lack of LYVE-1 expression and absence of apparent sprouting reaction when inflammatory lymphangiogenesis was induced from limbal lymphatics. The suture placement model used in this study is a relatively mild stimulus of inflammation. It offers us an ideal tool to identify the difference between limbal lymphatics and Schlemm's canal. It is possible that alternative methods with more robust stimulation may induce sprouting from the Schlemm's canal, which warrants further investigation.

In a previous ex vivo study on human donor eyes, it was indicated that lymphatic markers of Prox-1, LYVE-1 and podoplanin were not expressed in the Schlemm's canal [26]. In the current mouse study, we detected negative expression of LYVE-1 and podoplanin (data not shown) but positive expression of Prox-1 both in vivo and ex vivo. The in vivo live imaging technique has many advantages over ex vivo analysis. It allows for direct detection and visualization of the Prox-1^+^ structure at its natural location and physiological state. This in vivo method also eliminates possible morphological change or structural damage with ex vivo assays that require multiple processes of tissue sampling, fixation, and staining [16]. The current study should therefore provide more direct and accurate information on the expression pattern of Prox-1 in Schlemm's canal. Nevertheless, it is yet to be determined whether there is a discrepancy between Prox-1 expression in human and mouse Schlemm's canal, which is an unlikely case based on multiple studies on Prox-1 in other tissues and sites.

The significance of the study is threefold. First, the new finding that Schlemm's canal endothelium expressed a lymphatic specific marker further suggests its closer similarities with lymphatic endothelium than with blood endothelium. As summarized in Table 1 [6], [13], [23], [25]

**Table 1:** Expression of Endothelial Cell Markers [6], [13], [23], [25].

	Endothelium Cell Type		
Marker	Blood	Lymphatic	Schlemm's Canal
CD31	+	+	+
Prox-1	-	+	+
LYVE-1	-	+	-
			

(+) sign indicates positive expression and (−) means no detectable expression.

all three types of endothelium are known to express CD31 [23], [27]. However, the morphology of the canal endothelium more resembles that of the lymphatic endothelium in that they both have a discontinuous basement membrane with similar extracellular matrix support structures [3]. Interestingly, it is commonly accepted that both endothelia have similar vascular origins, with both differentiating from preexisting blood vascular endothelial cells during development [4], [6], [7]. There has been no evidence till now that the canal expresses a lymphatic specific marker. It is important to emphasize that the expressed marker, Prox-1, is a master control gene shown to drive the vascular endothelial differentiation into the lymphatic phenotype during development and maintain this phenotype during maturity. Understanding what role Prox-1 plays during the development and maintenance of the Schlemm's canal endothelium will be invaluable to our better understanding of this unique structure. It is possible that the vascular endothelial cells of the Schlemm's canal is programmed for Prox-1 expression as other typical lymphatics but is somehow arrested for subsequent expression of other lymphatic markers, such as LYVE-1, to achieve its unique features and distinctive functions in the outflow pathway, which requires further exploration.

Secondly, the panel of protein markers used in this study will allow researchers to better identify, isolate, and characterize Schlemm's canal endothelium. For example, research with Schlemm's canal endothelium has lagged behind that of trabecular meshwork cells due to lack of a distinguishing protein marker [28]. Our results suggest that sorting for CD31^+^, Prox-1^+^, and Lyve-1^−^ cells should provide a homogeneous population of Schlemm's canal endothelial cells (Table 1). Furthermore, this sorting method will simplify the exclusion criteria currently used for sorting Schlemm's canal endothelial cells and will result in better endothelial cell harvesting efficiency.

Lastly, we have provided a new model for Schlemm's canal research with the Prox-1-GFP transgenic mouse. Needless to say, live imaging has many advantages over conventional ex vivo investigation with dead tissues. This in vivo model for visualizing and studying Schlemm's canal is a new tool that we hope will allow researchers to study normal and pathological aqueous outflow in real time. It should also help researchers to better understand the canal's relationship with the lymphatic system and to what extent the lymphatic system determines intraocular pressure. Humans and mice share many common features of the aqueous outflow system more than those of rabbits and primates [29]. Thus, we believe that there is great potential that novel findings arising from this mouse model will someday translate into human clinical therapy for the treatment of ocular diseases associated with aqueous outflow, most importantly glaucoma.

## Supporting Information

Figure S1 **Cross-sectional immunohistochemical analysis of the iridocorneal angle of 3 week-old Prox-1 GFP mice.** (**A**) Representative images showing the Prox1^+^LYVE-1^−^Schlemm's canal (red asterisk) at the corneal scleral junction. Blue: DAPI for nuclear staining; Red: LYVE-1; Green: Prox-1. (**B**) Representative images showing both limbal lymphatics (white asterisk) and Schlemm's canal (red asterisk) expressed CD31. Blue: DAPI; Red: CD31; Green: Prox-1. Scale bars, 50 µm (A and B). SC, Schlemm's canal; Co, cornea; Cj, conjunctiva.
(TIF)

Figure S2 **Cross-sectional immunohistochemical analysis of the iridocorneal angle of 3 week-old wildtype mice.** (A) Representative images showing that Schlemm's canal (red asterisk) is Prox-1^+^ and CD31^+^. Blue: DAPI for nuclear staining; Red: Prox-1; Green: CD31. (**B**) Representative images showing CD31^+^ Schlemm's canal (red asterisk) is LYVE-1^−^. Blue: DAPI; Red: LYVE-1; Green: CD31^+^. Scale bars, µm (A and B). SC, Schlemm's canal; Co, cornea; Cj, conjunctiva.
(TIF)

Figure S3 **Fluorescent microscopic evaluation of sprouting lymphatic vessels into inflamed Prox-1 GFP mouse cornea after suture placement.** (**A**) Frontal view of the whole cornea showing lymphatic vessels encroaching towards the center. (**B**) Magnified view of boxed region in (A) showing that corneal lymphatics are emanating from limbal lymphatics. Yellow and white arrowhead corresponds to corneal and limbal lymphatics, respectively, in (A, C). (C) Side view of cornea providing further evidence that corneal lymphatics are sprouting from limbal vessels but not the more posterior Prox-1^+^ Schlemm's canal. Green: Prox-1. Scale bars, 500 µm (A); 250 µm (B and C).
(TIF)

Video S1 **Three-dimensional rotational view of irideocorneal angle together with the limbal area showing apparent sprouting of new lymphatic vessels from limbal lymphatics but not from Schlemm**'**s canal.** Green: Prox-1; Red: LYVE-1. Scale bar: 100 µm.
(WMV)
