# Supplementary figures and images for: Novel Characterization and Live Imaging of Schlemm's Canal Expressing Prox-1
Source: PLoS One. 2014 May 14;9(5):e98245. doi: 10.1371/journal.pone.0098245 (PMC4020937; doi:10.1371/journal.pone.0098245)

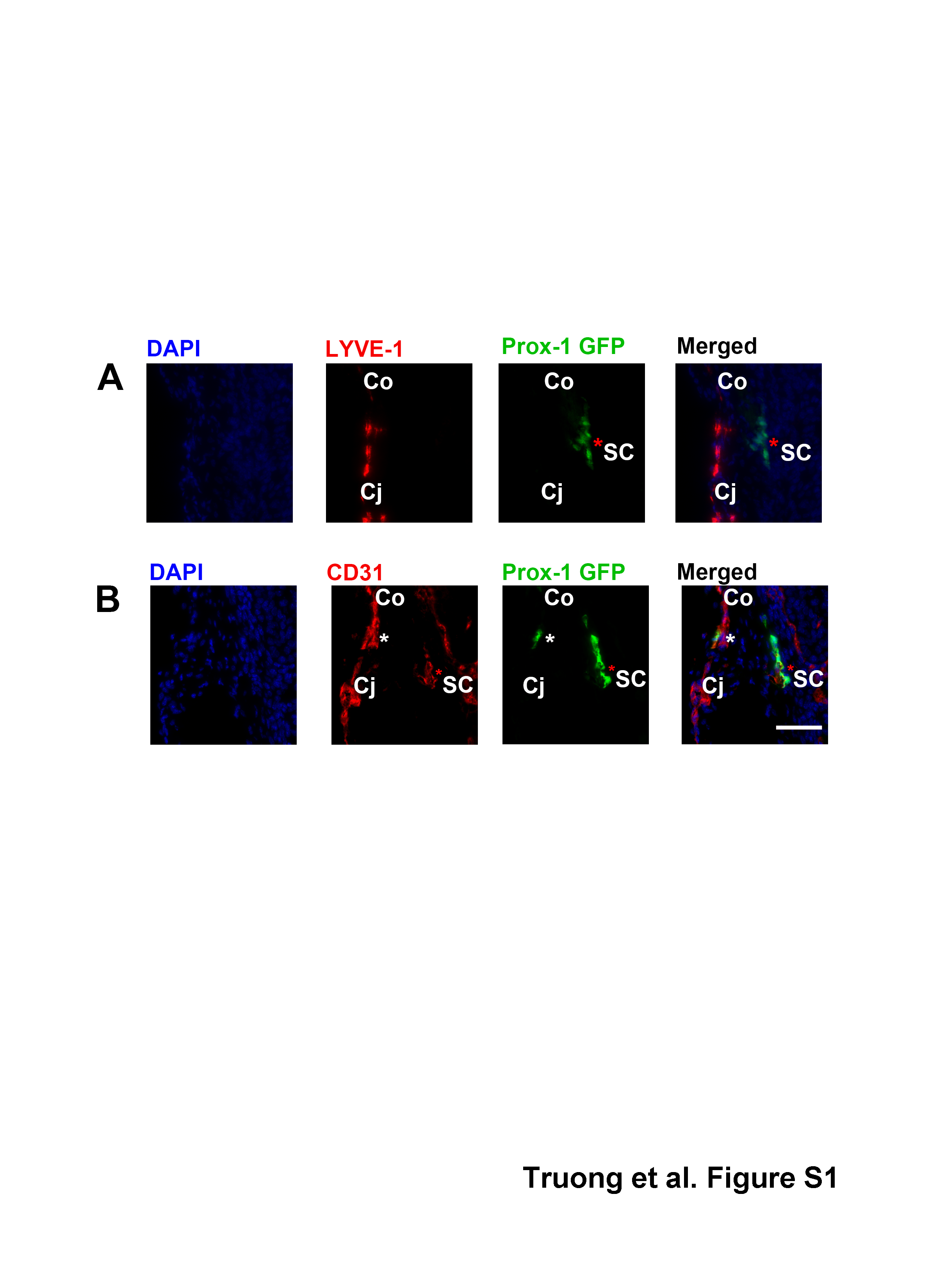

Supplement: Figure S1 — Cross-sectional immunohistochemical analysis of the iridocorneal angle of 3 week-old Prox-1 GFP mice. (A) Representative images showing the Prox1+LYVE-1−Schlemm's canal (red asterisk) at the corneal scleral junction. Blue: DAPI for nuclear staining; Red: LYVE-1; Green: Prox-1. (B) Representative images showing both limbal lymphatics (white asterisk) and Schlemm's canal (red asterisk) expressed CD31. Blue: DAPI; Red: CD31; Green: Prox-1. Scale bars, 50 µm (A and B). SC, Schlemm's canal; Co, cornea; Cj, conjunctiva. (TIF) [file pone.0098245.s001.tif]

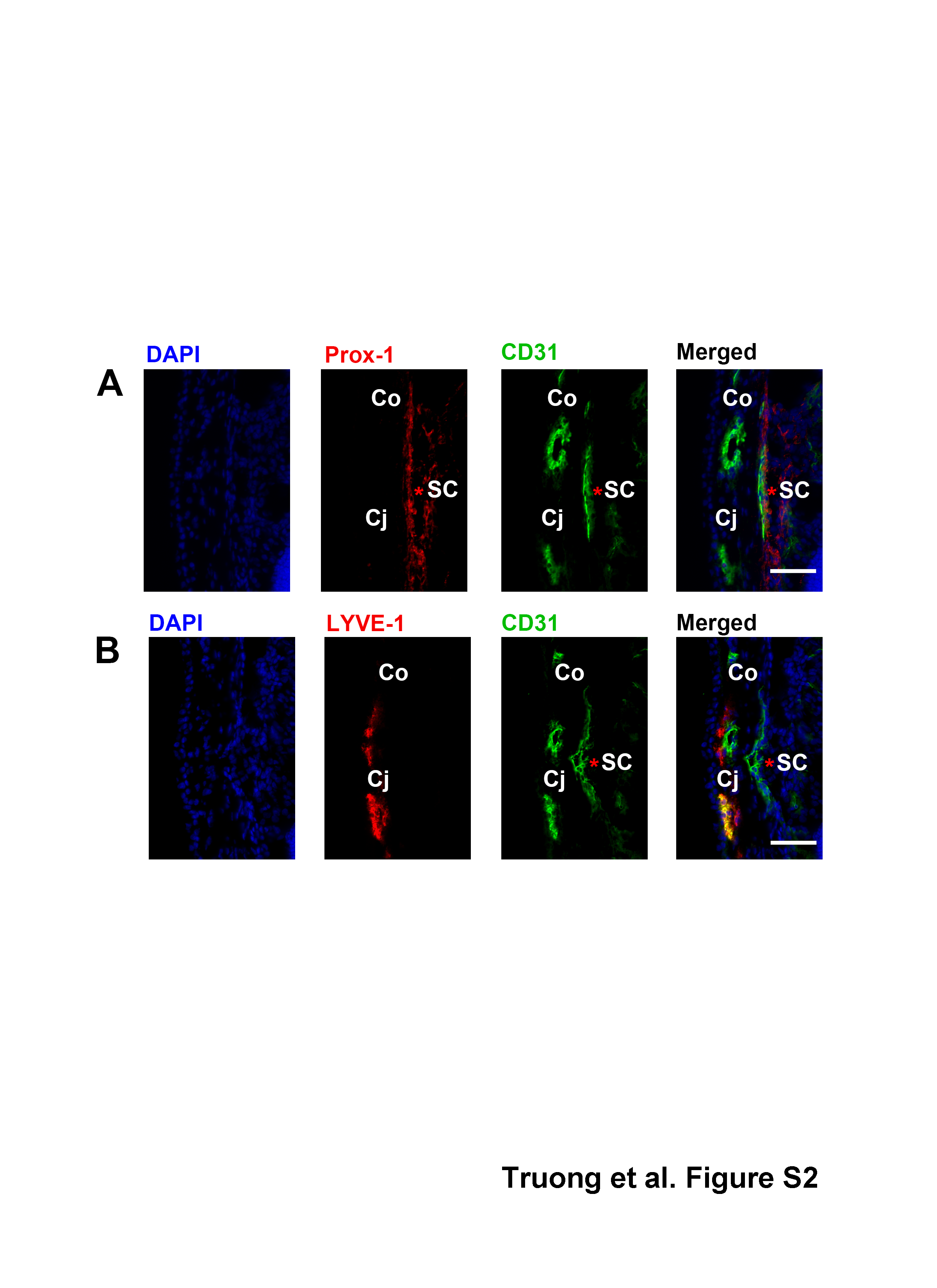

Supplement: Figure S2 — Cross-sectional immunohistochemical analysis of the iridocorneal angle of 3 week-old wildtype mice. (A) Representative images showing that Schlemm's canal (red asterisk) is Prox-1+ and CD31+. Blue: DAPI for nuclear staining; Red: Prox-1; Green: CD31. (B) Representative images showing CD31+ Schlemm's canal (red asterisk) is LYVE-1−. Blue: DAPI; Red: LYVE-1; Green: CD31+. Scale bars, µm (A and B). SC, Schlemm's canal; Co, cornea; Cj, conjunctiva. (TIF) [file pone.0098245.s002.tif]

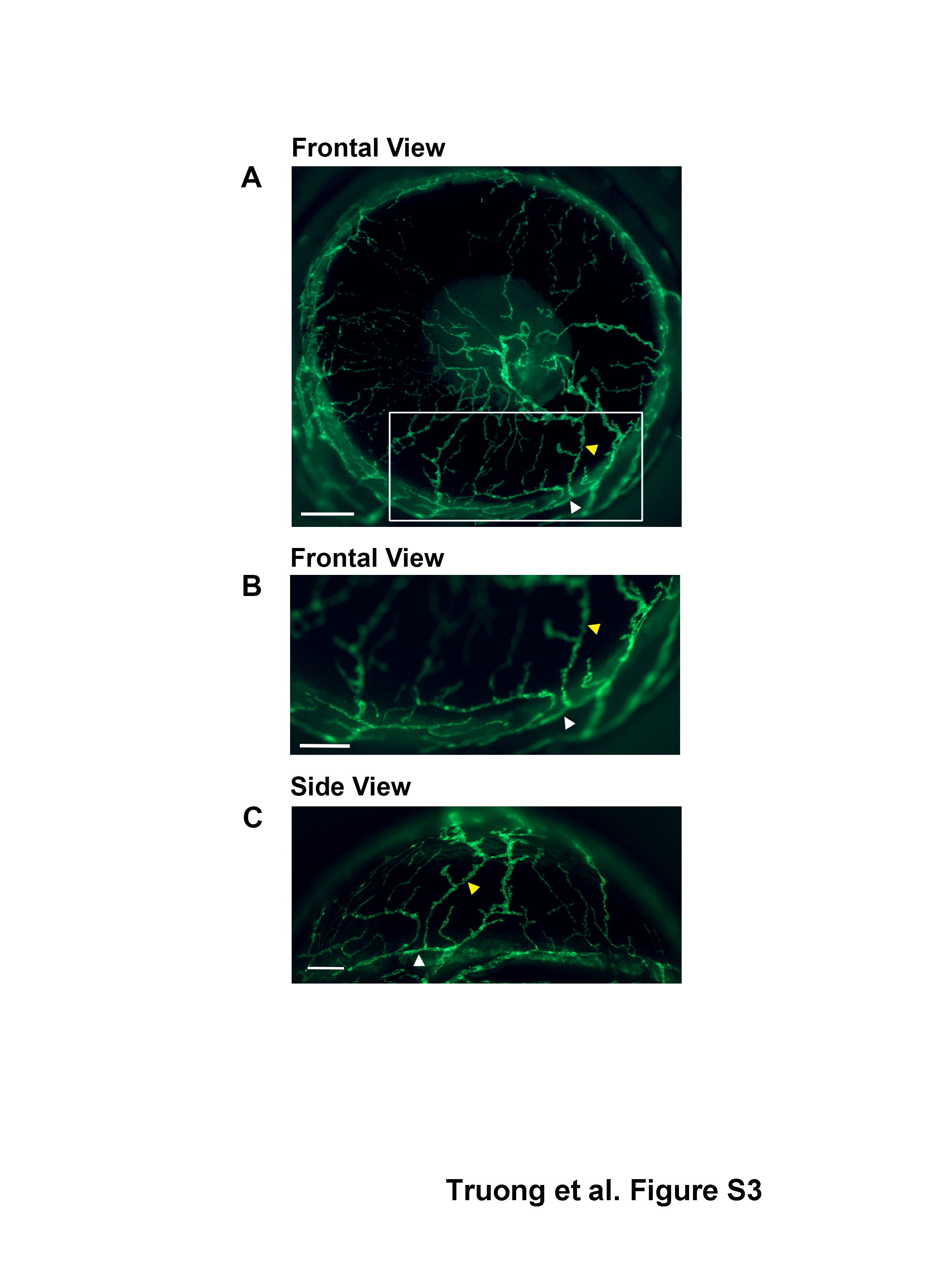

Supplement: Figure S3 — Fluorescent microscopic evaluation of sprouting lymphatic vessels into inflamed Prox-1 GFP mouse cornea after suture placement. (A) Frontal view of the whole cornea showing lymphatic vessels encroaching towards the center. (B) Magnified view of boxed region in (A) showing that corneal lymphatics are emanating from limbal lymphatics. Yellow and white arrowhead corresponds to corneal and limbal lymphatics, respectively, in (A, C). (C) Side view of cornea providing further evidence that corneal lymphatics are sprouting from limbal vessels but not the more posterior Prox-1+ Schlemm's canal. Green: Prox-1. Scale bars, 500 µm (A); 250 µm (B and C). (TIF) [file pone.0098245.s003.tif]
